# Supplementary material for: Modeling glioblastoma heterogeneity as a dynamic network of cell states
Source: Mol Syst Biol. 2021 Sep 16;17(9):e10105. doi: 10.15252/msb.202010105 (PMC8444284; doi:10.15252/msb.202010105)
Supplement: Supplementary file 6 — Source Data for Figure 5 [file MSB-17-e10105-s004.zip › Figure5A_sourcedata/GSEA_3071/nosigdb_stateII.GseaPreranked.1622795151171/RICHARDS_NOWAKOWSKI_SCIENCE_2017_NEN_EARLY2_UPREG.html]

Details for gene set RICHARDS\_NOWAKOWSKI\_SCIENCE\_2017\_NEN\_EARLY2\_UPREG[GSEA]

|  || Dataset | state23071 |
| Phenotype | NoPhenotypeAvailable |
| Upregulated in class | na\_neg |
| GeneSet | RICHARDS\_NOWAKOWSKI\_SCIENCE\_2017\_NEN\_EARLY2\_UPREG |
| Enrichment Score (ES) | -0.47267616 |
| Normalized Enrichment Score (NES) | -3.0020244 |
| Nominal p-value | 0.0 |
| FDR q-value | 0.0 |
| FWER p-Value | 0.0 |
Table: GSEA Results Summary

  

Fig 1: Enrichment plot: RICHARDS\_NOWAKOWSKI\_SCIENCE\_2017\_NEN\_EARLY2\_UPREG      
 Profile of the Running ES Score & Positions of GeneSet Members on the Rank Ordered List

  

| PROBE | GENE SYMBOL | GENE\_TITLE | RANK IN GENE LIST | RANK METRIC SCORE | RUNNING ES | CORE ENRICHMENT || 1 | LBH |  |  | 182 | 0.898 | -0.0401 | No |
| 2 | TLE3 |  |  | 215 | 0.861 | -0.0419 | No |
| 3 | SMURF2 |  |  | 224 | 0.851 | -0.0375 | No |
| 4 | ZFP30 |  |  | 272 | 0.782 | -0.0437 | No |
| 5 | EDIL3 |  |  | 331 | 0.727 | -0.0532 | No |
| 6 | NT5C3A |  |  | 359 | 0.705 | -0.0548 | No |
| 7 | EZR |  |  | 466 | 0.642 | -0.0773 | No |
| 8 | DDX10 |  |  | 537 | 0.609 | -0.0907 | No |
| 9 | NTM |  |  | 592 | 0.582 | -0.1003 | No |
| 10 | SMS |  |  | 608 | 0.575 | -0.0998 | No |
| 11 | SNRNP40 |  |  | 609 | 0.574 | -0.0954 | No |
| 12 | THBS1 |  |  | 624 | 0.568 | -0.0948 | No |
| 13 | HS6ST1 |  |  | 645 | 0.553 | -0.0957 | No |
| 14 | SLC38A1 |  |  | 656 | 0.550 | -0.0942 | No |
| 15 | NETO2 |  |  | 740 | 0.519 | -0.1116 | No |
| 16 | PAK1 |  |  | 781 | 0.505 | -0.1181 | No |
| 17 | CLMP |  |  | 822 | 0.490 | -0.1247 | No |
| 18 | PRDM8 |  |  | 846 | 0.486 | -0.1270 | No |
| 19 | CHD3 |  |  | 912 | 0.468 | -0.1402 | No |
| 20 | EIF2B3 |  |  | 945 | 0.460 | -0.1450 | No |
| 21 | SPATS2 |  |  | 989 | 0.447 | -0.1527 | No |
| 22 | KPNA3 |  |  | 1046 | 0.429 | -0.1639 | No |
| 23 | DGCR8 |  |  | 1122 | 0.414 | -0.1801 | No |
| 24 | RCC2 |  |  | 1124 | 0.413 | -0.1772 | No |
| 25 | CNKSR2 |  |  | 1156 | 0.406 | -0.1822 | No |
| 26 | TCERG1 |  |  | 1300 | 0.378 | -0.2162 | No |
| 27 | EFNB2 |  |  | 1306 | 0.377 | -0.2146 | No |
| 28 | NRP1 |  |  | 1372 | 0.362 | -0.2287 | No |
| 29 | WWC1 |  |  | 1384 | 0.360 | -0.2288 | No |
| 30 | L1CAM |  |  | 1402 | 0.357 | -0.2305 | No |
| 31 | GOLGA8A |  |  | 1526 | 0.335 | -0.2596 | No |
| 32 | ZNF566 |  |  | 1640 | 0.318 | -0.2864 | No |
| 33 | MTF2 |  |  | 1710 | 0.309 | -0.3018 | No |
| 34 | PKNOX1 |  |  | 1711 | 0.309 | -0.2995 | No |
| 35 | PSMG2 |  |  | 1723 | 0.307 | -0.3000 | No |
| 36 | LRPPRC |  |  | 1836 | 0.294 | -0.3267 | No |
| 37 | ZPR1 |  |  | 1842 | 0.293 | -0.3257 | No |
| 38 | ARPC5 |  |  | 1847 | 0.293 | -0.3245 | No |
| 39 | SLIT2 |  |  | 1873 | 0.289 | -0.3288 | No |
| 40 | CACNA2D1 |  |  | 1878 | 0.289 | -0.3277 | No |
| 41 | BZW2 |  |  | 1902 | 0.285 | -0.3314 | No |
| 42 | QSER1 |  |  | 1922 | 0.282 | -0.3342 | No |
| 43 | PPP2R1B |  |  | 1927 | 0.282 | -0.3331 | No |
| 44 | FRMD4A |  |  | 2092 | 0.263 | -0.3734 | No |
| 45 | MAPRE2 |  |  | 2138 | 0.258 | -0.3830 | No |
| 46 | LARP1 |  |  | 2193 | 0.253 | -0.3951 | No |
| 47 | DOCK4 |  |  | 2230 | -0.251 | -0.4024 | No |
| 48 | ZNF160 |  |  | 2236 | -0.252 | -0.4018 | No |
| 49 | AHDC1 |  |  | 2252 | -0.254 | -0.4038 | No |
| 50 | PTPRS |  |  | 2253 | -0.254 | -0.4019 | No |
| 51 | GNAI1 |  |  | 2254 | -0.254 | -0.3999 | No |
| 52 | DYNC1I2 |  |  | 2260 | -0.255 | -0.3993 | No |
| 53 | VASH1 |  |  | 2275 | -0.256 | -0.4010 | No |
| 54 | ZC2HC1A |  |  | 2277 | -0.257 | -0.3993 | No |
| 55 | ZNF195 |  |  | 2298 | -0.258 | -0.4025 | No |
| 56 | NEUROD1 |  |  | 2312 | -0.261 | -0.4039 | No |
| 57 | KIF3A |  |  | 2315 | -0.261 | -0.4024 | No |
| 58 | OCIAD2 |  |  | 2361 | -0.266 | -0.4120 | No |
| 59 | PPFIA2 |  |  | 2364 | -0.266 | -0.4105 | No |
| 60 | CAP2 |  |  | 2406 | -0.272 | -0.4190 | No |
| 61 | SEMA6D |  |  | 2407 | -0.272 | -0.4170 | No |
| 62 | VEZT |  |  | 2424 | -0.273 | -0.4190 | No |
| 63 | ARFGEF1 |  |  | 2425 | -0.273 | -0.4170 | No |
| 64 | LUC7L |  |  | 2430 | -0.275 | -0.4159 | No |
| 65 | ZBTB10 |  |  | 2459 | -0.279 | -0.4210 | No |
| 66 | MKLN1 |  |  | 2464 | -0.279 | -0.4199 | No |
| 67 | TM2D3 |  |  | 2494 | -0.285 | -0.4253 | No |
| 68 | LENG8 |  |  | 2507 | -0.286 | -0.4262 | No |
| 69 | SRGAP3 |  |  | 2508 | -0.286 | -0.4240 | No |
| 70 | CLASP2 |  |  | 2514 | -0.287 | -0.4232 | No |
| 71 | SPAST |  |  | 2544 | -0.293 | -0.4284 | No |
| 72 | RSBN1L |  |  | 2546 | -0.293 | -0.4265 | No |
| 73 | HIVEP3 |  |  | 2554 | -0.294 | -0.4261 | No |
| 74 | SLC44A5 |  |  | 2557 | -0.294 | -0.4243 | No |
| 75 | RSF1 |  |  | 2562 | -0.295 | -0.4231 | No |
| 76 | FAM32A |  |  | 2588 | -0.299 | -0.4273 | No |
| 77 | SORBS1 |  |  | 2639 | -0.307 | -0.4379 | No |
| 78 | TSPAN14 |  |  | 2643 | -0.307 | -0.4364 | No |
| 79 | CBFA2T2 |  |  | 2646 | -0.308 | -0.4345 | No |
| 80 | LONRF1 |  |  | 2668 | -0.312 | -0.4376 | No |
| 81 | IGF1R |  |  | 2700 | -0.316 | -0.4432 | No |
| 82 | GNG4 |  |  | 2702 | -0.316 | -0.4411 | No |
| 83 | TSPAN13 |  |  | 2706 | -0.318 | -0.4394 | No |
| 84 | SOX12 |  |  | 2721 | -0.320 | -0.4406 | No |
| 85 | NHSL1 |  |  | 2735 | -0.324 | -0.4415 | No |
| 86 | CEP170 |  |  | 2737 | -0.324 | -0.4394 | No |
| 87 | SLC29A4 |  |  | 2741 | -0.325 | -0.4377 | No |
| 88 | TRAK1 |  |  | 2784 | -0.332 | -0.4460 | No |
| 89 | SECISBP2 |  |  | 2802 | -0.335 | -0.4478 | No |
| 90 | GATAD2B |  |  | 2816 | -0.338 | -0.4486 | No |
| 91 | BRAF |  |  | 2825 | -0.341 | -0.4481 | No |
| 92 | CBLB |  |  | 2826 | -0.341 | -0.4455 | No |
| 93 | ATCAY |  |  | 2865 | -0.346 | -0.4527 | No |
| 94 | ZNF292 |  |  | 2882 | -0.352 | -0.4542 | No |
| 95 | ST3GAL6 |  |  | 2917 | -0.359 | -0.4602 | No |
| 96 | EVL |  |  | 2927 | -0.360 | -0.4598 | No |
| 97 | GTF2IRD2 |  |  | 2946 | -0.364 | -0.4617 | No |
| 98 | BSDC1 |  |  | 2961 | -0.366 | -0.4626 | No |
| 99 | ZNF286A |  |  | 2986 | -0.370 | -0.4660 | No |
| 100 | MLXIP |  |  | 2988 | -0.370 | -0.4634 | No |
| 101 | CPE |  |  | 2994 | -0.371 | -0.4619 | No |
| 102 | MIAT |  |  | 2996 | -0.371 | -0.4594 | No |
| 103 | ATP6V0A1 |  |  | 3014 | -0.378 | -0.4609 | No |
| 104 | TP53BP1 |  |  | 3024 | -0.378 | -0.4604 | No |
| 105 | ATXN7L3B |  |  | 3040 | -0.382 | -0.4613 | No |
| 106 | SLC22A17 |  |  | 3085 | -0.394 | -0.4697 | Yes |
| 107 | ZNF462 |  |  | 3086 | -0.394 | -0.4667 | Yes |
| 108 | KIF3C |  |  | 3087 | -0.394 | -0.4637 | Yes |
| 109 | CECR2 |  |  | 3089 | -0.395 | -0.4610 | Yes |
| 110 | PRKX |  |  | 3093 | -0.397 | -0.4588 | Yes |
| 111 | ENAH |  |  | 3136 | -0.410 | -0.4665 | Yes |
| 112 | TGFBR1 |  |  | 3139 | -0.411 | -0.4639 | Yes |
| 113 | PHF20L1 |  |  | 3143 | -0.412 | -0.4616 | Yes |
| 114 | DCC |  |  | 3155 | -0.415 | -0.4613 | Yes |
| 115 | CDH4 |  |  | 3164 | -0.418 | -0.4602 | Yes |
| 116 | NDUFA13 |  |  | 3190 | -0.426 | -0.4634 | Yes |
| 117 | C14orf132 |  |  | 3206 | -0.430 | -0.4640 | Yes |
| 118 | CHRDL1 |  |  | 3219 | -0.432 | -0.4638 | Yes |
| 119 | NARF |  |  | 3223 | -0.434 | -0.4613 | Yes |
| 120 | RTN1 |  |  | 3232 | -0.436 | -0.4601 | Yes |
| 121 | SOX5 |  |  | 3237 | -0.437 | -0.4578 | Yes |
| 122 | ZBTB18 |  |  | 3239 | -0.438 | -0.4548 | Yes |
| 123 | NAV1 |  |  | 3250 | -0.441 | -0.4540 | Yes |
| 124 | SDCBP |  |  | 3257 | -0.442 | -0.4522 | Yes |
| 125 | FYN |  |  | 3262 | -0.444 | -0.4499 | Yes |
| 126 | CYFIP2 |  |  | 3265 | -0.445 | -0.4470 | Yes |
| 127 | LDLRAD4 |  |  | 3271 | -0.448 | -0.4449 | Yes |
| 128 | CASC15 |  |  | 3308 | -0.460 | -0.4507 | Yes |
| 129 | CREBBP |  |  | 3329 | -0.466 | -0.4524 | Yes |
| 130 | ELAVL3 |  |  | 3341 | -0.472 | -0.4516 | Yes |
| 131 | BRD3 |  |  | 3346 | -0.474 | -0.4491 | Yes |
| 132 | RABGAP1 |  |  | 3347 | -0.475 | -0.4455 | Yes |
| 133 | PPP1R9A |  |  | 3366 | -0.486 | -0.4465 | Yes |
| 134 | B4GALT5 |  |  | 3372 | -0.487 | -0.4441 | Yes |
| 135 | PDE9A |  |  | 3396 | -0.496 | -0.4463 | Yes |
| 136 | STX6 |  |  | 3412 | -0.503 | -0.4463 | Yes |
| 137 | EIF1B |  |  | 3420 | -0.509 | -0.4443 | Yes |
| 138 | NUDT4 |  |  | 3421 | -0.510 | -0.4404 | Yes |
| 139 | KIDINS220 |  |  | 3459 | -0.523 | -0.4460 | Yes |
| 140 | KLF3 |  |  | 3481 | -0.533 | -0.4474 | Yes |
| 141 | SVIL |  |  | 3490 | -0.537 | -0.4454 | Yes |
| 142 | ADD2 |  |  | 3492 | -0.538 | -0.4416 | Yes |
| 143 | CALCOCO1 |  |  | 3512 | -0.548 | -0.4423 | Yes |
| 144 | ARHGAP21 |  |  | 3538 | -0.564 | -0.4445 | Yes |
| 145 | CTTNBP2 |  |  | 3541 | -0.565 | -0.4408 | Yes |
| 146 | ZNF506 |  |  | 3549 | -0.570 | -0.4383 | Yes |
| 147 | CELSR2 |  |  | 3550 | -0.570 | -0.4340 | Yes |
| 148 | HUNK |  |  | 3569 | -0.582 | -0.4342 | Yes |
| 149 | CRMP1 |  |  | 3583 | -0.594 | -0.4331 | Yes |
| 150 | FOXO3 |  |  | 3594 | -0.601 | -0.4311 | Yes |
| 151 | FOXG1 |  |  | 3604 | -0.607 | -0.4288 | Yes |
| 152 | AGO1 |  |  | 3611 | -0.609 | -0.4258 | Yes |
| 153 | FNBP1L |  |  | 3615 | -0.614 | -0.4219 | Yes |
| 154 | EPHA3 |  |  | 3616 | -0.614 | -0.4173 | Yes |
| 155 | PHLDA1 |  |  | 3622 | -0.617 | -0.4139 | Yes |
| 156 | CD24 |  |  | 3624 | -0.620 | -0.4094 | Yes |
| 157 | KIF5A |  |  | 3631 | -0.627 | -0.4063 | Yes |
| 158 | ANTXR1 |  |  | 3634 | -0.630 | -0.4020 | Yes |
| 159 | DPYSL5 |  |  | 3668 | -0.659 | -0.4055 | Yes |
| 160 | FOXN3 |  |  | 3671 | -0.661 | -0.4010 | Yes |
| 161 | RB1CC1 |  |  | 3681 | -0.669 | -0.3983 | Yes |
| 162 | IP6K2 |  |  | 3689 | -0.672 | -0.3950 | Yes |
| 163 | MCF2L |  |  | 3691 | -0.673 | -0.3902 | Yes |
| 164 | PREX1 |  |  | 3716 | -0.698 | -0.3911 | Yes |
| 165 | GSE1 |  |  | 3720 | -0.699 | -0.3866 | Yes |
| 166 | IRGQ |  |  | 3721 | -0.700 | -0.3813 | Yes |
| 167 | ZKSCAN1 |  |  | 3736 | -0.711 | -0.3795 | Yes |
| 168 | KLHL24 |  |  | 3743 | -0.723 | -0.3756 | Yes |
| 169 | GTF2I |  |  | 3749 | -0.727 | -0.3714 | Yes |
| 170 | ZNF704 |  |  | 3750 | -0.729 | -0.3659 | Yes |
| 171 | TAGLN3 |  |  | 3751 | -0.729 | -0.3604 | Yes |
| 172 | TOP2B |  |  | 3777 | -0.767 | -0.3610 | Yes |
| 173 | PLCB1 |  |  | 3779 | -0.769 | -0.3555 | Yes |
| 174 | TNRC6B |  |  | 3781 | -0.771 | -0.3499 | Yes |
| 175 | GRIA2 |  |  | 3786 | -0.774 | -0.3451 | Yes |
| 176 | ENC1 |  |  | 3787 | -0.774 | -0.3392 | Yes |
| 177 | NREP |  |  | 3805 | -0.794 | -0.3376 | Yes |
| 178 | ZNF608 |  |  | 3810 | -0.798 | -0.3326 | Yes |
| 179 | KIF5C |  |  | 3813 | -0.800 | -0.3271 | Yes |
| 180 | BICD1 |  |  | 3814 | -0.801 | -0.3210 | Yes |
| 181 | ST8SIA1 |  |  | 3815 | -0.801 | -0.3150 | Yes |
| 182 | MYEF2 |  |  | 3818 | -0.807 | -0.3094 | Yes |
| 183 | MAP3K13 |  |  | 3826 | -0.813 | -0.3050 | Yes |
| 184 | PGM2L1 |  |  | 3843 | -0.832 | -0.3029 | Yes |
| 185 | MEX3A |  |  | 3866 | -0.867 | -0.3020 | Yes |
| 186 | GRIA3 |  |  | 3870 | -0.879 | -0.2961 | Yes |
| 187 | TSC22D1 |  |  | 3872 | -0.887 | -0.2897 | Yes |
| 188 | ARIH1 |  |  | 3873 | -0.890 | -0.2829 | Yes |
| 189 | ROBO2 |  |  | 3879 | -0.899 | -0.2774 | Yes |
| 190 | IGSF3 |  |  | 3886 | -0.912 | -0.2721 | Yes |
| 191 | DDAH2 |  |  | 3895 | -0.936 | -0.2671 | Yes |
| 192 | TIAM2 |  |  | 3910 | -0.965 | -0.2634 | Yes |
| 193 | EML6 |  |  | 3938 | -1.035 | -0.2625 | Yes |
| 194 | MEIS3 |  |  | 3951 | -1.060 | -0.2576 | Yes |
| 195 | PDP1 |  |  | 3954 | -1.075 | -0.2500 | Yes |
| 196 | HES6 |  |  | 3961 | -1.092 | -0.2433 | Yes |
| 197 | AFF3 |  |  | 3964 | -1.099 | -0.2355 | Yes |
| 198 | FRMD4B |  |  | 3972 | -1.116 | -0.2288 | Yes |
| 199 | SOX11 |  |  | 3977 | -1.147 | -0.2212 | Yes |
| 200 | TNIK |  |  | 3979 | -1.157 | -0.2127 | Yes |
| 201 | PGAP1 |  |  | 3981 | -1.164 | -0.2042 | Yes |
| 202 | APP |  |  | 3982 | -1.164 | -0.1954 | Yes |
| 203 | CXADR |  |  | 3991 | -1.197 | -0.1884 | Yes |
| 204 | TTC28 |  |  | 3994 | -1.224 | -0.1796 | Yes |
| 205 | CSRP2 |  |  | 4000 | -1.238 | -0.1716 | Yes |
| 206 | GPM6A |  |  | 4002 | -1.248 | -0.1624 | Yes |
| 207 | NFIX |  |  | 4004 | -1.262 | -0.1531 | Yes |
| 208 | TUBA1A |  |  | 4006 | -1.302 | -0.1435 | Yes |
| 209 | NFIB |  |  | 4007 | -1.310 | -0.1336 | Yes |
| 210 | TUBB3 |  |  | 4009 | -1.334 | -0.1238 | Yes |
| 211 | PTPRO |  |  | 4011 | -1.344 | -0.1139 | Yes |
| 212 | KDM5B |  |  | 4016 | -1.364 | -0.1046 | Yes |
| 213 | SLC1A2 |  |  | 4040 | -1.555 | -0.0988 | Yes |
| 214 | POU3F3 |  |  | 4049 | -1.649 | -0.0884 | Yes |
| 215 | MAP2 |  |  | 4051 | -1.673 | -0.0760 | Yes |
| 216 | PLXNA4 |  |  | 4060 | -1.752 | -0.0648 | Yes |
| 217 | SORL1 |  |  | 4062 | -1.787 | -0.0515 | Yes |
| 218 | TUBB2B |  |  | 4063 | -1.810 | -0.0379 | Yes |
| 219 | POU3F2 |  |  | 4071 | -2.024 | -0.0244 | Yes |
| 220 | BEX1 |  |  | 4072 | -2.034 | -0.0090 | Yes |
| 221 | MEIS2 |  |  | 4073 | -2.041 | 0.0064 | Yes |
Table: GSEA details [plain text format]

  

Fig 2: RICHARDS\_NOWAKOWSKI\_SCIENCE\_2017\_NEN\_EARLY2\_UPREG: Random ES distribution      
 Gene set null distribution of ES for **RICHARDS\_NOWAKOWSKI\_SCIENCE\_2017\_NEN\_EARLY2\_UPREG**
